# Effect of Coronal Alignment on 10-Year Survivorship of a Single Contemporary Total Knee Arthroplasty

**DOI:** 10.3390/jcm10010142

**Published:** 2021-01-04

**Authors:** Meagan E. Tibbo, Afton K. Limberg, Kevin I. Perry, Mark W. Pagnano, Michael J. Stuart, Arlen D. Hanssen, Matthew P. Abdel

**Affiliations:** Department of Orthopedic Surgery, Mayo Clinic, Rochester, MN 55905, USA; tibbo.meagan@mayo.edu (M.E.T.); limberg.afton@mayo.edu (A.K.L.); perry.kevin@mayo.edu (K.I.P.); pagnano.mark@mayo.edu (M.W.P.); stuart.michael@mayo.edu (M.J.S.); hanssen.arlen@mayo.edu (A.D.H.)

**Keywords:** mechanical axis, coronal alignment, total knee arthroplasty, survivorship

## Abstract

Debate remains regarding the utility of mechanical axis alignment as a predictor of durability after total knee arthroplasty (TKA). Our study aimed to assess the effects of coronal alignment on implant durability, clinical outcomes, and radiographic results with a single fixed-bearing TKA design. All patients undergoing primary cemented TKA of a single design (Stryker Triathlon) from 2005–2007 with >10 years of follow-up and available pre-operative and post-operative hip–knee–ankle radiographs were included (*n* = 89). Radiographs were measured to determine coronal alignment and assessed for loosening. Mean preoperative mechanical axis alignment was −6° ± 6.7° (varus, range, −16°–23°), while mean post-operative alignment was −1° ± 2.7° (varus, range, −3°–15°). The aligned group was defined as knees with a post-operative mechanical axis of 0° ± 3° (*n* = 73) and the outlier group as those outside this range (*n* = 16). No patients underwent revision. Ten-year survivorship free from any reoperation was 99% and 100% in the aligned and outlier groups, respectively (*p* = 0.64). Knee Society scores improved significantly in both groups (*p* < 0.001) and did not differ at final follow-up (*p* = 0.15). No knees demonstrated radiographic evidence of loosening. Post-operative mechanical axis alignment within 3° of neutral was not associated with improved implant durability, clinical outcomes, or radiographic results at 10 years following primary TKA.

## 1. Introduction

With the advent of precise and accurate surgical technology, there has been renewed interest in the effect of coronal plane alignment on clinical, radiographic, and patient-reported outcome measures (PROMs) following primary total knee arthroplasty (TKA) [1,2]. Proponents of mechanical alignment have previously suggested that correcting coronal plane alignment to within 0° ± 3° of the mechanical axis improves implant durability [3,4,5,6,7,8]. Significant healthcare resources have been devoted to utilizing computer-based navigation and robotic systems to provide surgeons with the ability to maintain this narrow margin of error [1,9,10,11,12,13,14]. Many of these technologies have been successful in increasing the precision of coronal plane alignment [1,2,15].

Concurrently, there has been continued discussion surrounding the use of non-traditional alignment targets in an effort to improve patient function without compromising implant durability [16,17]. There are few studies which report long-term outcomes of “kinematic alignment” [18,19,20,21,22]. However, a recent randomized clinical trial (RCT) did not demonstrate any significant clinical difference in 5-year PROMs, pain, function, or radiographic differences when comparing mechanical and kinematic alignment techniques [5,23]. Numerous other studies have investigated the impact of mechanical axis alignment outside of 0° ± 3° in the long-term [3,24,25], but most have included a variety of implants with many no longer considered contemporary.

The aim of the current study is to determine if there is a difference in implant survivorship, clinical outcomes, and radiographic results with a single design contemporary TKA frequently used in current clinical practice based upon a neutral mechanical axis (defined as 0° ± 3°) vs. those knees outside that range. We hypothesize that survivorship as well as clinical and radiographic outcomes will differ among patients with neutral mechanical axis alignment compared to those who fall outside of this target range.

## 2. Experimental Section

### 2.1. Patients

A retrospective review of our total joint registry (TJR) identified all patients who underwent a primary TKA with a single design at our institution between 2005 and 2007 by two high volume surgeons. A total of 3552 primary TKAs were performed at our institution between 2005 and 2007. Clinical and radiographic data were collected for 164 patients (187 TKAs). Seventy-eight patients (98 TKAs) were excluded due to lack of standing hip–knee–ankle radiographs. The inclusion criteria were intentionally strict: (1) treatment with a single-design primary TKA, (2) use of a posterior-stabilized tibial insert, (3) patellar resurfacing with an all-polyethylene (PE) patellar component, (4) cement fixation for all components, (5) a minimum potential follow-up of 10 years defined as a study interval between 2005 and 2007, and (6) availability of preoperative and post-operative standing hip–knee–ankle radiographs obtained according to a previously published protocol [26], 86 patients (89 TKAs) met criteria, including 47 with conventional PE and 42 with highly cross-linked PE. Institutional review board (IRB) approval was obtained prior to initiation of the study.

The final cohort of 86 patients (89 TKAs) had a mean age at TKA of 65 years (range, 32–83 years) and mean body mass index (BMI) of 34 kg/m^2^ (range, 22–57 kg/m^2^). There were 56 females (65%) and 33 males (35%). Mean follow-up was 10 years (range, 10–12 years). The aligned group had a mean age of 64 years (range, 32–82 years) and the outlier group had a mean age of 67 years (range, 51–83 years; *p* = 0.36). The mean BMI in the aligned and outlier groups was 34 kg/m^2^ (range, 22–57 kg/m^2^) and 34 kg/m^2^ (range, 24–43 kg/m^2^; *p* = 0.23). There were 48 females and 25 males in the aligned group, and 8 females and 8 males in the outlier group (*p* = 0.26).

### 2.2. Surgical Technique and Indications

Indications for the procedure were primary osteoarthritis (*n* = 77), post-traumatic arthritis (*n* = 10), rheumatoid arthritis (*n* = 1), and avascular necrosis (*n* = 1). One total knee arthroplasty design was evaluated: Triathlon Total Knee System (Stryker; Mahwah, New Jersey). All procedures were performed by high-volume hip and knee arthroplasty surgeons at a single academic institution. A medial parapatellar arthrotomy was used in all cases. Component positioning was performed using an intramedullary guide on the femoral side and an extramedullary guide for the tibia. The surgeon’s definitive goal in all cases was to obtain neutral mechanical axis alignment post-operatively. Coronal plane deformities were corrected first using bony resection, and second with appropriate soft-tissue releases as needed to create rectangular gaps in flexion and extension and throughout an entire arc of motion. Manual instrumentation was utilized in all cases.

### 2.3. Radiographic Analysis

**Figure 1:**
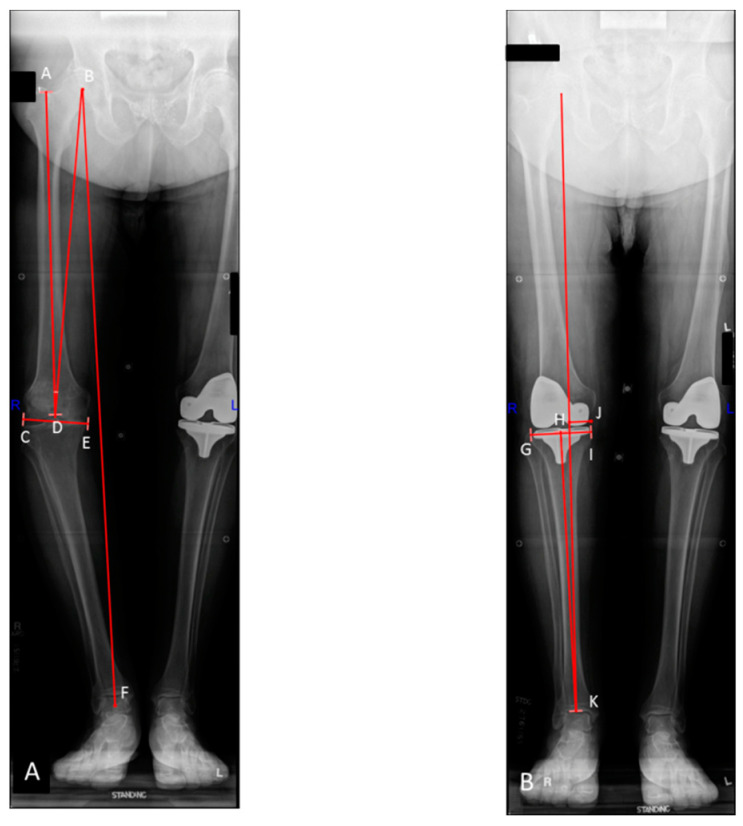
Preoperative (**A**) and post-operative (**B**) standing hip–knee–ankle radiographs following primary total knee arthroplasty (TKA) of patient in the outlier group. Line B–F represents the mechanical axis of the limb. The angle formed by lines A–D and C–E represents the mechanical axis of the femur. Angle A–D–B represents the femoral mechanical axis (FMA) angle. The angle formed by lines H–K and G–I represents the mechanical axis of the tibia. The distance from point J to the mechanical axis of the limb represents the post-operative mechanical width.

Preoperative and post-operative hip–knee–ankle radiographs were obtained according to a previously published, standardized protocol [26]. Radiographs were obtained within 2 months prior to surgery and within 3 months post-operatively. Lower limb mechanical axis alignment, femoral and tibial angles, as well as the distance from the mechanical axis to the center of the knee were measured by two independent observers (M.E.T. and A.K.L.) using the criteria described by Cooke et al. [27,28,29]. All measurement conflicts were resolved via consensus between observers at the time of review. Valgus alignment was denoted using positive values, while negative values represented varus alignment. Plateau width was defined as the maximal medial-lateral dimension of the proximal tibia at the knee joint line. Tibial slope was measured on standing lateral radiographs in a standardized fashion. Mechanical width was defined as the distance from the medial tibial plateau to the mechanical axis of the limb (Figure 1A,B). Radiographic evidence of loosening or mechanical failure was independently assessed by 3 additional high-volume arthroplasty surgeons (A.D.H., K.I.P., M.P.A.).

**Figure 2:**
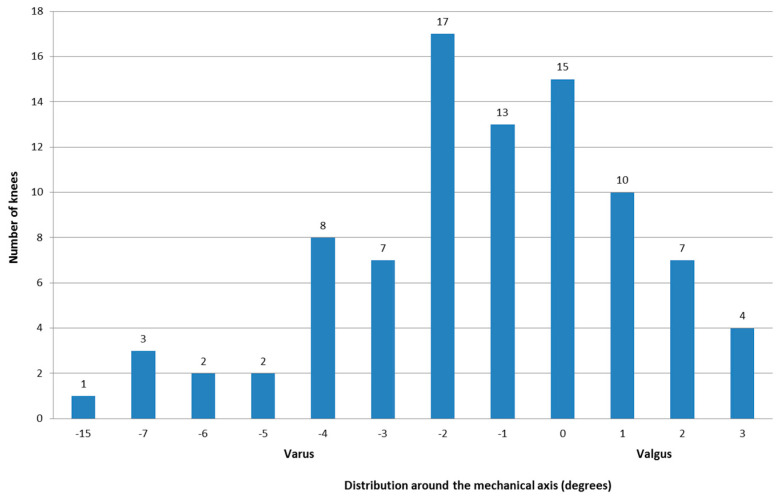
Bar graph depicting the distribution of the post-operative alignment around the mechanical axis. Negative values indicate varus alignment, and positive values represent valgus alignment.

Mean preoperative mechanical limb alignment was −5.7° in the aligned group and −8.1° in the outlier group (*p* = 0.20). Mean post-operative mechanical limb alignment was −0.5° in the aligned group and −5.5° in the outlier group (*p* < 0.001, Figure 2). All additional mean preoperative and post-operative alignment measurements can be found in Table 1.

### 2.4. Clinical Outcomes

Our institutional TJR was used to collect demographic information as well as data on revisions, reoperations, and complications. Standard clinical and radiographic follow-up was obtained at 3 months, 6 months, 1 year, 2 years, and every 5 years thereafter. All total joint arthroplasty patients at our institution completed a standardized patient questionnaire at every follow-up visit with appropriate radiographs. At our institution, the decision regarding whether to obtain pre- and post-operative full-length standing radiographs is based on surgeon preference. The aforementioned questionnaires were used to assess Knee Society scores (KSS) preoperatively and post-operatively [30]. Additional data detailing the reasons for revision or reoperation were extracted directly from the medical record via detailed chart review.

### 2.5. Statistical Methods

Data acquisition and analysis were performed in compliance with protocols approved by the Mayo Clinic Institutional Review Board (ethical approval number 17-010458). Written informed consent was obtained from all participants prior to study initiation. To assess the effect of mechanical alignment on implant survivorship and clinical outcomes, we divided the cohort into two groups: (1) a mechanically aligned group (mechanical axis 180° ± 3° which results in a mechanical alignment of 0° ± 3°), and (2) an outlier group (mechanical axis <177° or >183° which results in a mechanical alignment outside of 0° ± 3°). The Kaplan-Meier method [31] was used to create survivorship curves at 10 years for both groups with endpoints including any revision, aseptic revision, and any reoperation. Differences in Knee Society scores (KSS) were determined by comparing scores in the aligned group to scores in the outlier group at the latest follow-up for all patients using the student’s t-test. Those who experienced death, revision, or reoperation were censored at the visit prior to the event. All statistical tests were two-sided and a *p* value <0.05 was considered significant. All statistical analyses were performed utilizing GraphPad Prism version 7.03 for Windows.

## 3. Results

### 3.1. Implant Survivorship

There were no revisions in the mechanically aligned or outlier group at 10 years post-operatively. One patient in the aligned group underwent reoperation for hematoma evacuation and wound revision 18 days post-operatively. As such, the 10-year survivorship free from any reoperation was 99% and 100% in the aligned and outlier groups, respectively (*p* = 0.64). One patient in both the aligned and outlier groups underwent manipulation under anesthesia for stiffness (*p* = 0.26).

### 3.2. Clinical Outcomes

Knee Society scores improved significantly (*p* < 0.001) in the aligned group from 32 preoperatively to 91, 92, and 92 at 2, 5, and 10 years, respectively (Table 2). A similar trend was noted in the outlier group in which KSS improved from 34 preoperatively to 89, 89, and 94 at 2, 5, and 10 years, respectively. Knee Society scores did not differ between groups at final follow-up (*p* = 0.15).

### 3.3. Radiographic Results

One knee in the aligned group demonstrated progressive subsidence of the tibial component at 10 years. The patient was a 55-year-old male with a BMI of 41 kg/m^2^ at the time of the index primary TKA. However, a revision arthroplasty has not yet been performed due to his lack of symptoms. No other knees demonstrated radiographic evidence of loosening at last follow up. No knees in either group had evidence of polyethylene wear or periprosthetic osteolysis at last follow up.

## 4. Discussion

In the present series describing a single, contemporary TKA design, survivorship free from revision was 100% at 10 years for knees in both the aligned and outlier groups. Neutral mechanical axis alignment after total knee arthroplasty is a widely accepted tenet due to the plethora of historical data supporting this target to improve implant durability. Data demonstrating better function, however, is lacking. The dearth of data in this realm is the driving force behind investigations of non-traditional alignment targets. A variety of technologies in contemporary practice allow the surgeon to effectively hit any target reliably and reproducibly. It is reasonable, therefore, to further investigate non-traditional alignment targets, but is best done in contemporary implants with known track records.

We were also unable to identify any difference in revision or reoperation between groups. These results support findings of prior large series from our institution which did not demonstrate a survival advantage for knees in the aligned group when compared to the outlier group at 5, 10, or 20 years post-operatively [3,24]. That being said, knees in the outlier group for those studies, as well as the current study, were only mildly outside the range of 0° ± 3°. Similarly, Howell et al. [21] reported 98% survivorship free from any revision and 98% free from aseptic failure at 10 years in their series of kinematically-aligned knees using a single prosthesis design, implanted using patient-specific instrumentation. A recently published systematic review and meta-analysis comparing mechanically and kinematically aligned TKAs found no difference in complications, reoperations, or need for revision surgery in five RCTs directly comparing the two techniques [32]. Our findings are in agreement with those of the aforementioned authors. However, there remains a need for long-term survivorship data directly comparing outcomes of TKA performed using mechanical and non-traditional alignment targets. To our knowledge, the present series is one of the largest in current literature describing the durability of a single, contemporary, frequently utilized implant design at 10 years.

Clinical results in our study paralleled survivorship findings demonstrating statistically significant improvements, though no significant clinical difference in KSS between aligned and outlier groups at latest follow-up. KSS improved from 32 in the aligned group and 34 in the outlier group preoperatively to 92 and 94 at 10 years of follow-up, respectively. These results mirror previously published outcomes from our institution; however, this research remains vital since there continues to be a subset of patients with well-aligned and well-fixed TKAs who are unhappy with their result [3,24]. A recent systematic review of 13 studies comparing anatomic, adjusted mechanical, kinematic, and restricted kinematic alignment techniques found that kinematic alignment, for slight to mid-range coronal plane deformity, led to faster recovery and higher functional outcomes when compared to mechanical alignment [20]. The studies included in the above systematic review utilized WOMAC, SF-36, OKS, EQ-5D, KOOS, UCLA, and TUG tests to assess function. These scores may be more sensitive and less likely to be limited by the ceiling phenomenon known to constrain the utility of KSS assessed herein. As our ability to more precisely attain target alignment improves, future studies will need to better elucidate subtle differences in outcome across alignment techniques. We need to focus on sensitive functional outcome metrics like those listed above and potentially novel ones including the Forgotten Joint Score (FJS) [33].

With respect to our third aim, assessment of radiographic outcome, we found no difference in the rate of radiographic loosening between aligned and outlier knees. Analogous to results from Abdel et al. [3], Parratte et al. [24], and others [21], we did not identify significant radiographic evidence of loosening, polyethylene wear, or osteolysis at 10 years following primary TKA with this single implant. Historically, supporters of neutral mechanical alignment have cited concerns for loosening if prostheses were aligned outside of the 0° ± 3° range [6,7,8,34]. Our data suggest that alignment slightly outside of that range is well-tolerated and does not lead to increased risk of aseptic loosening at 10 years.

The present study is not without limitations. First, the retrospective, single-center review focused on a single, contemporary prosthesis and as such results may not be generalizable to all practice settings or implant designs. However, these strict inclusion criteria increase the internal validity of our results and allowed for significantly greater standardized measurement of a multiplicity of radiographic measurements over a period of decade. Second, hip–knee–ankle radiographs were obtained based on the discretion of the treating surgeon, which may introduce unique biases. The availability of hip–knee–ankle radiographs was also one of the exclusion criteria for the study. Thirdly, more recent studies have demonstrated that three dimensional imaging may increase the accuracy of limb alignment measurements. Three dimensional imaging was not available in our cohort which may limit the accuracy of our measurements. Finally, the clinical outcome score utilized, KSS, suffers from a ceiling effect when patient outcomes are excellent. Future studies would benefit from the use of more discerning functional outcome tools to identify subtle difference between groups. Additionally, due to the small number of patients in the outlier group, we may not have been able to identify a significant difference between two groups.

## 5. Conclusions

In conclusion, post-operative mechanical axis alignment within 3° of neutral was not associated with improved implant durability, clinical outcomes, or radiographic results in this series at 10 years following primary total knee arthroplasty. Small deviations from neutral mechanical alignment with respected boundaries (e.g., 2° or 3° of varus) are likely to be well tolerated by this particular prosthesis.

## Figures and Tables

**Table 1:** Mean ± standard deviation of pre- and post-operative radiographic measurements among aligned and outlier TKAs.

	Aligned	Outlier	Overall
Preoperative Measurements *			
Mechanical Femur	1.0 ± 3.1	0.8 ± 2.6	1.0 ± 3.0
FMA Angle	−6.0 ± 0.8	−6.5 ± 1.3	−6.1 ± 0.9
TMA	−2.9 ± 3.7	−4.5 ± 2.6	−3.3 ± 3.6
Overall Mechanical	−5.7 ± 6.9	−8.1 ± 6.0	−6.2 ± 6.7
Plateau W (mm)	83.1 ± 7.1	85.5 ± 9.5	83.5 ± 7.6
Mechanical W (mm)	21.1 ± 25.4	12.0 ± 14.5	19.4 ± 24.7
Tibial Slope	6.2 ± 3.9	7.5 ± 4.0	6.4 ± 3.9
Post-operative Measurements *			
Mechanical Femur	0.3 ± 1.3	−0.3 ± 2.4	0.2 ± 1.6
Mechanical Tibia	−0.5 ± 1.6	−5.5 ± 2.7	−1.4 ± 2.7
Overall Mechanical	74.7 ± 4.7	77.8 ± 7.0	75.3 ± 5.2
Plateau W (mm)	35.0 ± 6.1	27.0 ± 14.1	33.6 ± 8.6
Mechanical W (mm)	12 ± 3.3	11.5 ± 3.3	11.9 ± 3.3
Tibial Slope	1.7 ± 2.8	2.1 ± 2.9	1.7 ± 2.8

* Degrees, FMA = femoral mechanical axis, W = width, TMA = tibial mechanical angle.

**Table 2:** Mean, standard deviation and range of clinical outcomes.

	Aligned	Outlier
**Pre-op**		
Knee Society scores	32.1 ± 21.1 (0.0–92.0)	33.8 ± 11.0 (19.0–43.0)
Function Score	55.8 ± 16.5 (20.0–100.0)	53.2 ± 15.4 (30.0–80.0)
Pain Score	16.5 ± 10.8 (0.0–50.0)	17.9 ± 9.8 (0.0–30.0)
**2 Year Post-op**		
Knee Society Score	90.7 ± 6.0 (74.0–95.0)	88.9 ± 7.5 (73.0–95.0)
Function Score	81.9 ± 18.7 (40.0–100.0)	64.0 ± 21.3 (30.0–100.0)
Pain Score	48.2 ± 5.0 (20.0–50.0)	46.3 ± 7.7 (20.0–50.0)
**5 Year Post-op**		
Knee Society Score	92.0 ± 3.9 (80.0–95.0)	89.4 ± 5.9 (78.0–94.0)
Function Score	78.0 ± 21.1 (20.0–100.0)	76.2 ± 30.3 (0.0–100.0)
Pain Score	48.3 ± 5.5 (10.0–50.0)	46.2 ± 11.0 (10.0–50.0)
**10 Year Post-op**		
Knee Society Score	92.4 ± 4.3 (74.0–95.0)	93.7 ± 0.6 (93.0–94.0)
Function Score	70.5 ± 25.4 (0.0–100.0)	45.0 ± 31.3 (0.0–100.0)
Pain Score	48.3 ± 6.1 (10.0–50.0)	45.4 ± 9.1 (20.0–50.0)

## Data Availability

The data presented in this study are available on request from the corresponding author. The data are not publicly available due to Institutional Review Board requirements.

## References

[1] Mannan A., Vun J., Lodge C., Eyre-Brook A., Jones S. (2018). Increased precision of coronal plane outcomes in robotic-assisted total knee arthroplasty: A systematic review and meta-analysis. Surgeon.

[2] Liow M.H., Xia Z., Wong M.K., Tay K.J., Yeo S.J., Chin P.L. (2014). Robot-assisted total knee arthroplasty accurately restores the joint line and mechanical axis. A prospective randomised study. J. Arthroplast..

[3] Abdel M.P., Ollivier M., Parratte S., Trousdale R.T., Berry D.J., Pagnano M.W. (2018). Effect of Postoperative Mechanical Axis Alignment on Survival and Functional Outcomes of Modern Total Knee Arthroplasties with Cement: A Concise Follow-up at 20 Years. J. Bone Jt. Surg. Am..

[4] Insall J.N., Hood R.W., Flawn L.B., Sullivan D.J. (1983). The total condylar knee prosthesis in gonarthrosis. A five to nine-year follow-up of the first one hundred consecutive replacements. J. Bone Jt. Surg. Am..

[5] Young S.W., Walker M.L., Bayan A., Briant-Evans T., Pavlou P., Farrington B. (2017). The Chitranjan S. Ranawat Award: No Difference in 2-year Functional Outcomes Using Kinematic versus Mechanical Alignment in TKA: A Randomized Controlled Clinical Trial. Clin. Orthop. Relat. Res..

[6] Bargren J.H., Blaha J.D., Freeman M.A. (1983). Alignment in total knee arthroplasty. Correlated biomechanical and clinical observations. Clin. Orthop. Relat. Res..

[7] Hsu R.W., Himeno S., Coventry M.B., Chao E.Y. (1990). Normal axial alignment of the lower extremity and load-bearing distribution at the knee. Clin. Orthop. Relat. Res..

[8] Ritter M.A., Faris P.M., Keating E.M., Meding J.B. (1994). Postoperative alignment of total knee replacement. Its effect on survival. Clin. Orthop. Relat. Res..

[9] Jones C.W., Jerabek S.A. (2018). Current Role of Computer Navigation in Total Knee Arthroplasty. J. Arthroplast..

[10] Matassi F., Cozzi Lepri A., Innocenti M., Zanna L., Civinini R., Innocenti M. (2019). Total Knee Arthroplasty in Patients with Extra-Articular Deformity: Restoration of Mechanical Alignment Using Accelerometer-Based Navigation System. J. Arthroplast..

[11] Urish K.L., Conditt M., Roche M., Rubash H.E. (2016). Robotic Total Knee Arthroplasty: Surgical Assistant for a Customized Normal Kinematic Knee. Orthopedics.

[12] Yang H.Y., Seon J.K., Shin Y.J., Lim H.A., Song E.K. (2017). Robotic Total Knee Arthroplasty with a Cruciate-Retaining Implant: A 10-Year Follow-up Study. Clin. Orthop. Surg..

[13] Van der List J.P., Chawla H., Joskowicz L., Pearle A.D. (2016). Current state of computer navigation and robotics in unicompartmental and total knee arthroplasty: A systematic review with meta-analysis. Knee Surg. Sports Traumatol. Arthrosc..

[14] Cho K.J., Seon J.K., Jang W.Y., Park C.G., Song E.K. (2019). Robotic versus conventional primary total knee arthroplasty: Clinical and radiological long-term results with a minimum follow-up of ten years. Int. Orthop..

[15] Moon Y.W., Ha C.W., Do K.H., Kim C.Y., Han J.H., Na S.E., Lee C.H., Kim J.G., Park Y.S. (2012). Comparison of robot-assisted and conventional total knee arthroplasty: A controlled cadaver study using multiparameter quantitative three-dimensional CT assessment of alignment. Comput. Aided Surg..

[16] Blakeney W., Clément J., Desmeules F., Hagemeister N., Rivière C., Vendittoli P.A. (2019). Kinematic alignment in total knee arthroplasty better reproduces normal gait than mechanical alignment. Knee Surg. Sports Traumatol. Arthrosc..

[17] An V.V.G., Twiggs J., Leie M., Fritsch B.A. (2019). Kinematic alignment is bone and soft tissue preserving compared to mechanical alignment in total knee arthroplasty. Knee.

[18] Li Y., Wang S., Wang Y., Yang M. (2018). Does Kinematic Alignment Improve Short-Term Functional Outcomes after Total Knee Arthroplasty Compared with Mechanical Alignment? A Systematic Review and Meta-analysis. J. Knee Surg..

[19] Oussedik S., Abdel M.P., Victor J., Pagnano M.W., Haddad F.S. (2020). Alignment in total knee arthroplasty. Bone Jt. J..

[20] Rivière C., Iranpour F., Auvinet E., Howell S., Vendittoli P.A., Cobb J., Parratte S. (2017). Alignment options for total knee arthroplasty: A systematic review. Orthop. Traumatol. Surg. Res..

[21] Howell S.M., Shelton T.J., Hull M.L. (2018). Implant Survival and Function Ten Years After Kinematically Aligned Total Knee Arthroplasty. J. Arthroplast..

[22] Howell S.M., Papadopoulos S., Kuznik K., Ghaly L.R., Hull M.L. (2015). Does varus alignment adversely affect implant survival and function six years after kinematically aligned total knee arthroplasty?. Int. Orthop..

[23] Young S.W., Sullivan N.P.T., Walker M.L., Holland S., Bayan A., Farrington B. (2020). No Difference in 5-year Clinical or Radiographic Outcomes Between Kinematic and Mechanical Alignment in TKA: A Randomized Controlled Trial. Clin. Orthop. Relat. Res..

[24] Parratte S., Pagnano M.W., Trousdale R.T., Berry D.J. (2010). Effect of postoperative mechanical axis alignment on the fifteen-year survival of modern, cemented total knee replacements. J. Bone Jt. Surg. Am..

[25] Bonner T.J., Eardley W.G., Patterson P., Gregg P.J. (2011). The effect of post-operative mechanical axis alignment on the survival of primary total knee replacements after a follow-up of 15 years. J. Bone Jt. Surg. Br..

[26] McGrory J.E., Trousdale R.T., Pagnano M.W., Nigbur M. (2002). Preoperative hip to ankle radiographs in total knee arthroplasty. Clin. Orthop. Relat. Res..

[27] Cooke T.D., Scudamore R.A., Bryant J.T., Sorbie C., Siu D., Fisher B. (1991). A quantitative approach to radiography of the lower limb. Principles and applications. J. Bone Jt. Surg. Br..

[28] Cooke T.D., Sled E.A., Scudamore R.A. (2007). Frontal plane knee alignment: A call for standardized measurement. J. Rheumatol..

[29] Cooke T.D. (2002). Definition of axial alignment of the lower extremity. J. Bone Jt. Surg. Am..

[30] Harris W.H. (1969). Traumatic arthritis of the hip after dislocation and acetabular fractures: Treatment by mold arthroplasty. An end-result study using a new method of result evaluation. J. Bone Jt. Surg. Am..

[31] Goel M.K., Khanna P., Kishore J. (2010). Understanding survival analysis: Kaplan-Meier estimate. Int. J. Ayurveda Res..

[32] Takahashi T., Ansari J., Pandit H.G. (2018). Kinematically Aligned Total Knee Arthroplasty or Mechanically Aligned Total Knee Arthroplasty. J. Knee Surg..

[33] Carlson V.R., Post Z.D., Orozco F.R., Davis D.M., Lutz R.W., Ong A.C. (2018). When Does the Knee Feel Normal Again: A Cross-Sectional Study Assessing the Forgotten Joint Score in Patients After Total Knee Arthroplasty. J. Arthroplast..

[34] Lotke P.A., Ecker M.L. (1977). Influence of positioning of prosthesis in total knee replacement. J. Bone Jt. Surg. Am..

